# Zur Herstellung von Selbsthilfe-Räumen: Interaktionsdynamiken in Selbsthilfegruppen

**DOI:** 10.1007/s12054-022-00516-w

**Published:** 2022-09-30

**Authors:** Vera Dangel, Lukas Baumann, Brigitte Anderl-Doliwa

**Affiliations:** 1 Hochschule RheinMain, Wiesbaden, Deutschland; 2 Universität Klagenfurt, Klagenfurt, Österreich; 3 Katholische Hochschule, Mainz, Deutschland

**Keywords:** Selbsthilfe, Räume, Atmosphären, Emotionen, Strategische Aneignung

## Abstract

Der Beitrag diskutiert teilnehmend beobachtete Interaktionsdynamiken in Online-Selbsthilfegruppen zu Zeiten von Corona. Orientiert am Datenmaterial werden von den Teilnehmenden hervorgebrachte „Räume“ entlang der Thematiken „Atmosphäre“, „Gefühle“ und „strategische Aneignung“ vorgestellt. Die Analyse zeigt die Potenziale dialogisch ausgerichteter Begegnungsformate auf, welche eine von Solidarität geprägte Auseinandersetzung mit biographischen Verwundungen eröffnen und die Teilnehmenden als handelnde Subjekte in ihrer Begegnung mit als einschränkend wahrgenommenen sozialen Umweltbedingungen bestärken.

Wie gestalten sich Interaktionsdynamiken in Selbsthilfegruppen? Schlummert in der Idee der Selbsthilfe, als Interessenvereinigung von sich gegenseitig unterstützen Peers, eine Quelle für Veränderungsprozesse? Können professionell Handelnde hier etwas lernen, auch vor dem Hintergrund von Online-Treffen in Zeiten von Corona.

Im Zentrum dieses Beitrags stehen Interaktionsdynamiken von Peers in Selbsthilfegruppen, die im Rahmen des BMBF geförderten Praxisforschungsprojekts VISION-RA^1^ [1] teilnehmend beobachtet wurden. Das Erkenntnisinteresse von VISION-RA umschließt (misslingende) affektive Abstimmungsprozesse zwischen (gemeinde-)psychiatrischen Fachkräften und Nutzenden ihrer sozialen Dienstleistungen in Alltagssituationen. Ausschlaggebend für die Erweiterung der Zusammenarbeit mit Vertreter_innen der Selbsthilfe war einerseits, dass im Frühjahr 2021 aufgrund der nationalen Corona-Pandemieentwicklungen eingeschränkte Zugangsregelungen zu den mit VISION-RA kooperierenden gemeindepsychiatrischen Einrichtungen bestanden und die Forschenden daher keine durchgängige Datenerhebung durchführen konnten. Andererseits war nicht nur die Gestaltung von Beziehungsverhältnissen zwischen Peers im Kontrast zu professionellen Beziehungsverhältnissen von Forschungsinteresse, sondern auch die Analyse von Interaktionsdynamiken in Online-Formaten zu Zeiten von Corona. Bereits vorhandene Kontakte zu Selbsthilfegruppen eröffneten die vielversprechende Möglichkeit, Online-Treffen teilnehmend zu beobachten.

## Datengewinnung

In einer einführenden Online-Veranstaltung zum gegenseitigen Kennenlernen wurde die Vereinbarung zur teilnehmenden Beobachtung und digitalen Aufzeichnung von Online-Treffen getroffen. Bei der Beobachtung des ersten Online-Treffens kam auf Seiten der Forschenden der Eindruck auf, die Gruppendynamik zu irritieren. Wiederholt wurden ihnen u. a. biographische Zusammenhänge zu einzelnen Teilnehmenden ausführlich erläutert. In Rücksprache mit den Vertreter_innen der Selbsthilfe partizipierten die Forschenden infolge ohne Aktivierung ihrer Kamera und ihrem Mikrofon „still“ im Hintergrund, um ihren Einfluss auf die Gruppensituation der Online-Treffen möglichst gering zu halten.

## Analysevorgehen

Orientiert am Forschungsstil der von Strauss und Corbin (1996) vorgeschlagenen *Grounded Theory Methodologie *gingen wir die Durchsicht des Gesamtdatenkorpus, bestehend aus sechs digital aufgezeichneten Online-Treffen mit durchschnittlicher Dauer von jeweils ca. 60 min, an. Aus dem Datenmaterial traten die drei Dimensionen

1. Atmosphäre,
2. affektives Erleben und
3. strategische Aneignung

heraus. Im Zuge des analytischen „Aufbrechen[s] und Konzeptualisieren[s]“ (Strauss und Corbin 1996, S. 45) fokussierten wir, geleitet von der Datenmaterialdichte, jene sechs Online-Treffen.

## Selbsthilfe-Räume

In den untersuchten Selbsthilfegruppen werden von Teilnehmenden verschiedene Räume hergestellt, in denen vielfältige Bezüge ausgetauscht, fixiert und aufgelöst werden können. Diese in sich verschränkten Räume sind schwer voneinander abzugrenzen, jedoch weisen sie spezifische Eigensinnigkeiten auf, weshalb hier eine analytische Trennung als gewinnbringend erachtet wird.

Selbsthilfegruppen stellen Räume dar, in denen gemeinsam schwierige Lebenssituationen und Konflikte bewältigt sowie Informationen ausgetauscht werden können. Die Moderation der Selbsthilfegruppe bestätigt diese Aussage und beschreibt eine Funktion der Selbsthilfegruppe wie folgt: „Wir können einigen durch akute Krisen helfen und auch zur dauernden Stabilisierung beitragen“. Diese Funktion sehen auch Haller und Gräser (2012), sie beschreiben die Förderung seelischer Gesundheit als Zentrum von Selbsthilfegruppen. Bei der Frage, wie in Selbsthilfegruppen seelische Gesundheit gefördert wird, drängt sich der Begriff Selbsthilfe-Räume geradezu auf. Die Teilnehmenden begegnen sich mit Wohlwollen, wodurch geschützte Räume entstehen. Es geht aber nicht nur um schützen, sondern auch um stützen, manchmal auch um fordern und konfrontieren (Rockel und Semmler 2012). Es geht um Solidarität und Parteilichkeit zwischen Menschen, die Ähnliches erfahren haben und um das Teilen von gegenwärtigem Leid sowie biographischen Verwundungen. Es entstehen viele Räume, deren Gleichzeitigkeit ein wichtiges Strukturmoment der Selbsthilfe darstellt. Auch aus diesem Grund haben wir den Fokus der Selbsthilfe-Räume in unserem Datenmaterial gewählt. Die hier identifizierten Selbsthilfe Dimensionen weisen auf Möglichkeitsräume hin, die für Veränderungsprozesse bedeutsam werden können (Ehlers 2019).

## Der atmosphärische Raum

Die Teilnehmenden der Selbsthilfegruppen halten sich während der Sitzungen in Küchen, Wohn- und Arbeitszimmern, auf Terrassen oder auch in einer psychiatrischen Institutsambulanz auf. Dabei werden nicht nur die privaten Räumlichkeiten, sondern auch Alltagspraktiken wie Rauchen, Kaffeetrinken oder Essen geteilt. Humor, Redseligkeit und die schnelle Abfolge von Stimmungen erwecken den Eindruck einer dynamischen und geselligen digitalen Umgebung, was sich durch das Sprechen in starkem Dialekt sowie der etablierten Duz-Kultur weiter verstärkt. Der Reihe nach erzählen die Teilnehmenden von ihrer aktuellen Lebenssituation, beantworten und stellen Fragen und reagieren auf Kommentare aus der Gruppe. Als ebenfalls Betroffene steht die Moderatorin der Gruppen als verbindende Mitte im Raum, dabei nimmt sie wechselnde Funktionen ein – mal ist sie Anker für Teilnehmende in emotional herausfordernden Situationen, mal tritt sie als Fackelträgerin in Erscheinung, wobei sie nicht nur die Redebeiträge koordiniert, sondern auch Licht ins Dunkel bzw. Sprache ins Schweigen bringt. Stellvertretende und metaphorische Deutungen sowie positive Bestärkung sowohl der Moderatorin als auch der Teilnehmenden untereinander stellen eine Bedeutungspluralität entsprechend der eigenen Position her und ermöglichen eine kollektive Anteilnahme durch geteilte Erfahrung, was sich in den Worten, „Wo stehst Du gerade?“, „Du bist kein Einzelfall“, „Wir alle kennen das“, „Also weißt Du, Susanne*, ich sehe das so!“ ausdrückt. Gemeinsames Empören über subjektiv erlebte Ungerechtigkeiten hebt ein entstehendes Gruppen-Wir nochmals hervor. Das Oszillieren zwischen der Öffnung des subjektiv Privaten und dem Gruppenaustausch über Alltägliches wie das Wetter, Dating oder das Großeltern- bzw. Mutter-Sein erschafft so gewissermaßen eine geschützte intime Öffentlichkeit. Jene bezieht dabei nicht nur die virtuell Anwesenden mit ein, sondern auch Personen, die nicht anwesend sein können. So werden „Grüße aus der Backstube“ ausgerichtet, ein Kühlschrank wird verschenkt, es werden Umzugshelfer_innen angefragt, Know-How geteilt, gemeinsame Erlebnisse erinnert und ausstehende Treffen abseits des Online-Formats geplant. Die virtuelle Entgrenzung dieses hergestellten sozialen Raums erscheint vor dem Hintergrund des in der Corona-Pandemie geforderten „Social Distancing“ dabei paradox. Wo einerseits physische Begegnungen an materiellen Orten verunmöglicht werden, so kann die Verlagerung der Begegnung in digitale Räume Momente sozialer Nähe erzeugen. Wo die Teilnehmenden die eigenen Handlungschancen als begrenzt empfinden und den Wunsch nach physischen Begegnungen äußern, weil das als „ganz anders“ und „direkter“ empfunden wird, so wird gleichzeitig die Qualität des kontinuierlichen, empathischen und ungezwungenen Online-Austauschs „auf Augenhöhe“ als Ermöglichung sozialer Interaktion überhaupt hervorgehoben: „Besser hier als gar nicht treffen“, „also ich komme gerne her, sogar vom Krankenhaus aus“. Die Verschränkung vielfältiger örtlicher und zeitlicher Bezüge verdichtet die Selbsthilfegruppen einerseits zu einem atmosphärischen Raum sozialer Nähe und gleichzeitig weist er weit über ihn hinaus. Jene entstehende digitale *soziale Atmosphäre* (Gugutzer 2013, S. 305) kennzeichnet die Online-Selbsthilfegruppen so auf sehr spezifische Weise.

Im Kontrast zu professionellen psychiatrischen Settings, welche als „kalt“, „nüchtern“, „durchreguliert“ oder „weit weg vom Leben“ beschrieben werden, gestalten die Teilnehmenden einen Raum, in welchem sie ohne „Vorwurfshaltung“, „Leistungsdruck“ oder „Erfolgsforderungen“ „ernst- und wahrgenommen“ werden sowie gegenseitige Unterstützung sowie Verständnis erwarten und sich auf vielfältige Weise begegnen können. Durch den niedrigschwelligen Angebotscharakter und das ungezwungene Zusammensein verbinden sich die Teilnehmenden hinter ihren Bildschirmen an verschiedenen Orten auf leiblich-affektive Weise solidarisch miteinander. Konnektivität meint dann mehr als stabiles Internet, nämlich erwartbare soziale Nähe durch gemeinsames Wahrnehmen, Erinnern und Vorstellen – trotz und gerade wegen der physischen Distanz.

## Der Gefühlsraum

Die teilnehmende Karin^2^ besucht neben der Selbsthilfegruppe eine psychiatrische Tagesklinik. Dort finden regelmäßige Gruppengespräche mit Nutzenden und Fachkräften statt, sie schildert dieses Therapiegruppensystem auf Nachfrage der Teilnehmenden wie es ihr in der Tagesklinik ergehe, wie folgt: „Sie haben mir gesagt, ich wäre eine Schildkröte, man könne an meinen Panzer klopfen, ich ließe niemanden rein. Ich wäre nur traurig und ohne Lebensfreude, ich zöge die Gruppe runter“. Auf die Frage der anderen Teilnehmenden, wie sie sich dabei gefühlt habe, sagt sie: „Einerseits ist es mir egal, andererseits ist es nicht schön, wenn man andere runterzieht.“

Eine negative Bewertung sei schwer auszuhalten, bestätigen die Gruppenmitglieder und sagen einen Satz der immer, fast als Gruppenglaubenssatz, wiederholt wird: „Das geht mir ganz genauso“. Belastende, problematische Themen werden „vergemeinschaftet“. Daraufhin Karin nun mit kämpferisch wirkender Gestik: „In meinem Schildkrötenpanzer ist für solche Leute, die mich negativ bewerten, nicht viel Platz, wenn sie anklopfen.“ Und nun folgt eine Umdeutung des Schildkrötenpanzerbildes als Schutzraum und es entsteht Selbstwille: „Ich kann und will mich nicht ändern, nur damit die Anderen zufrieden sind.“ Die Angst vor Verletzung führt hier offensichtlich zur Verteidigung des Schutzpanzers.

Was sei das denn für ein Gefühl, fragen die Teilnehmenden wieder: „Andere würden wütend werden, aber mir ist das egal.“ Darauf Teilnehmerin Helga: „Mich macht es wütend, wenn man so mit dir umgeht.“ Augenscheinlich findet ein „stellvertretendes Fühlen“ statt begleitet von einem fragenden Hinweis auf eine biographische Verwundung: „Bist du vielleicht deshalb nicht wütend, weil du solche Situationen gut kennst?“ Daraufhin Karin: „Ja, wenn ich den Kopf rausstrecke, dann kriege ich immer eins drauf, daher brauche ich meinen Panzer. Ich bin stabil traurig, das hält mich von anderen fern, gibt mir Halt, Stärke und Schutz.“ Das negative Gefühl kann als Ressource „Schutzraum Panzer“ umgedeutet werden. Diese Umdeutung wird auch von den teilnehmenden aufgenommen, sie spiegeln: „Du bist eben authentisch, ehrlich und unverstellt, seit einem Jahr wiederholst du den selben Glaubenssatz – mein Leben ist mir scheißegal – uns hat das nie belastet, wir wissen wie das ist, wir sind ein Club bei dem jeder viel Leid erlebt hat.“ Hier wird deutlich, dass biographische Verwundungen und problematische Erfahrungen leichter thematisiert werden können, wenn sie geteilt werden. In der Gruppeninteraktion entsteht ein Raum, in dem Teilnehmende immer-neu-lernen, aus dem eigenen Erleben und Fühlen heraus und von dem Erlebten und Gefühlten Anderer. Es geht über die eigene Geschichte hinaus und wird genährt aus der Solidarität mit Menschen, die Ähnliches erfahren (Zwicknagel 2022).

Hier geht es auch um die Bewältigung des eigenen Leids, des eigenen Schicksals. Bei Karin durch Verachtung: „Mein Leben ist mir scheißegal“, ebenso wie Albert Camus, der im antiken Mythos des Sisyphos ein Symbol der Überwindung sieht. Indem es durch Verachtung überwunden wird, kann eine Überlegenheit über das eigene Schicksal hergestellt werden (Schumacher 2022). Oder eben durch „geteiltes Leid“ als Gruppenstrategie: „Wir sind ein Club, wo jeder viel erlebt hat.“ Ein weiterer auffälliger Aspekt der Interaktion im Gefühlsraum ist der, zum Teil sehr abrupte, Ebenenwechsel. Die Gruppe bohrt bei Karin immer wieder nach: „Wie fühlst du dich dabei“, dann Karin: „Ich will die Erinnerung nicht immer wieder hochholen, jetzt nicht, vielleicht ein anderes Mal.“ Daraufhin wechselt die Gruppe, ganz ohne Übergang, zu praktischen Alltagsthemen, hier, wie mit dem Einkaufen während der Pandemie sicher umzugehen ist. Nach einigen Minuten kommt dann plötzlich die Frage einer Teilnehmenden an Karin: „Was ist denn noch außer Traurigkeit und mein Leben ist mir scheißegal in dir?“ Daraufhin Karin: „Ich bin sehr gerne hier in der Gruppe.“ Dann wendet sich die Gruppe dem Thema einer anderen Teilnehmenden zu. Während die Ebenen der insistierenden Exploration der tiefen Ergründung von Emotionen dient, scheinen Themenwechsel hingegen entlastende Funktionen zu haben. Antworten dürfen dann für sich stehen bleiben, ohne zusätzliche Erläuterungen werden sie unhinterfragt aufgenommen.

## Raum zur strategischen Aneignung sozialer Umwelt

Wie in Selbsthilfegruppen dem Pandemiegeschehen begegnet wird, veranschaulichen die Teilnehmenden nicht nur beispielhaft vermittels der Reorganisation ihrer ursprünglich persönlichen Treffen hin zum online-Format. Neben jener konstruktiven Vorgehensweise mit der sie umgebenden materiellen Umwelt, nehmen sie die im Frühjahr 2021 national geltende Impfberechtigungsrangfolge spezifischer Statusgruppen zum Anlass, die Diskrepanz zwischen individuellen und gesellschaftlichen Interessen zu beleuchten und greifen damit auch Entwicklungen der sozialen Umwelt auf. Sie diskutieren die gegenwärtige „hohe Inzidenz“ in Verbindung mit Kontakteinschränkungen „am Arbeitsplatz“, „in der Schule“ sowie aus der Perspektive von „Großmüttern“ und stellen fest, dass „Schnelltests nicht zuverlässig [sind].“ Im Dialog entziffern sie (ihre) Infizierungsbedenken als Hauptursache und erkennen zugleich, dass ihnen „das Menschliche“, also die „stinknormalen Kontakte“, fehlen. Im Zuge des Zusammentragens von subjektiven Sichtweisen entwickeln die Teilnehmenden schließlich die kollektive Positionierung, dass die gegenwärtige Corona-Politik eine Schieflage prägt, da Impfzentren sogar offiziell anerkannte Impfberechtigte mit „achtwöchiger Wartezeit“ auf die Vakzination konfrontieren. Gestützt auf die in der Gruppe geteilte COVID-19-Impfbefürwortung erfolgt das Plädoyer, „die müssen die Priorisierung aufheben“, um sodann entlang von Gedankenexperimenten die Wunschoption „Impfen to go“ zur Befriedung des hierzulande von ihnen wahrgenommenen „Impfneids“ herauszustellen. Dass im dialogischen Austausch eine reflexive Distanz zu ihrem „Du bist so machtlos“ bzw. „wie in einem Käfig eingeschlossen“ umschriebenen Erleben aufgrund von Corona-Regeln erarbeitet wird und die Teilnehmenden sich damit einen interessensgeleiteten, kreativen Umgang mit ebendiesen Regeln der sozialen Umwelt eröffnen, offenbaren insbesondere die Reaktionsweisen auf die Unterstützung ersuchenden Hinweise zur Impfregistrierung der Teilnehmerin Rita. Ritas Artikulationen, sich mit Auskunft über ihre körperlichen Einschränkungen für eine COVID 19-Impfung registriert zu haben und mit Sorge um die Virusweitergabe an ihren über sechzigjährigen Ehemann eine lange Wartezeit zu befürchten, begegnen die Teilnehmenden mit Heranziehung der ihnen etikettierten biomedizinischen psychiatrischen Diagnosen. Zugunsten einer möglichst zügigen Impfterminvergabe an Rita durch dafür zuständig erklärte Autoritäten prüft die Gruppe eine „Modulation“ (Goffman 2018, S. 98) ebendieser menschliches Sozialverhalten kategorisierenden Regel-Richtlinie. So entziffern die Teilnehmenden die Reich- und Auslegungsweite der im Feld der (Sozial‑)Psychiatrie dominanten naturalisierenden Deutungsmuster zum Phänomen seelischer Gesundheit, indem sie sich gegenseitig informieren, dass die „Diagnosen, wie schwere Depressionen und schwere Schizophrenie“ auch COVID-19-Impfregistrierungen legitimieren.

Hinzufügend dechiffriert die Teilnehmende Luisa beispielhaft fachärztliche Handhabungsweisen der pathologisierenden biomedizinischen Kategoriesysteme mit dem Hinweis, „ich dachte, ein Attest gilt nur dann, wenn man in einer schweren Phase ist, das bin ich im Moment nicht, aber die [Psychiaterin, Verf.] hat mir das trotzdem ausgefüllt“. Die gewonnene Erkenntnis dient den Teilnehmenden zur Erörterung eines „strategische[n] Täuschungsmanöver[s]“ (Goffman 2018, S. 118). Damit stellen die Teilnehmenden einen Interessensausgleich in Bezug auf die gesellschaftlich vorherrschende traditionelle psychiatrische „Denkschablone von ‚wir‘ und ‚sie‘“ (Weinmann 2019, S. 33) her, welchen die Gruppenmoderation vermittels der Aufforderung, „Rita, guck’ doch mal, ob wir dich nicht mit Schizo durchkriegen“ pointiert zum Ausdruck bringt.

## Fazit

Was können professionell Handelnde von Interaktionsdynamiken in Selbsthilfegruppen lernen? Charakteristisch für die Selbsthilfe ist die Fokussierung auf biographische Verwundungen und die damit einhergehenden Emotionen. Diese werden, teilweise durch insistierende Explorationen, in das Zentrum der Interaktion gestellt. Auch die konsequente Ressourcenorientierung ist bedeutsam, kontrastierend zu der häufig defizitorientierten Ausrichtung psychiatrischer Institutionen überwiegt hier Hinwendung zu salutogenetischen Fragestellungen sowie eine Orientierung an den positiven Seiten der Person. Um von einer Demoralisation des Selbst (durch fortdauernde negative Erfahrung) zu einer Remoralisierung des Selbst zu gelangen, werden positive Affekte aktiviert und somit die Rückgewinnung von Handlungsfähigkeit ermöglicht. Hierbei spielen die Aktivierung und Nutzung von Ressourcen eine besondere Rolle.

Eine weitere Lesart erlaubt die Interaktionsdynamiken der Teilnehmenden auch vor dem Hintergrund einer „Aneignungsperspektive“ (Deinet und Reutlinger 2020, S. 1719) aufzufächern: In den die Selbsthilfegruppen kennzeichnenden dialogisch ausgerichteten online-Begegnungen „(…) werden menschliche Handlungen und Entwicklungsprozesse des Entzifferns, Erkennens, aber auch (neu) Gestaltens, Abänderns und Modifizierens der Umwelt (…)“ (ebd., S. 1720), in der die Teilnehmenden leben, sichtbar. Festhalten lässt sich demnach ein von Interaktionsdynamiken hervorgebrachter Raum, in welchem sich die Teilnehmenden mit als einschränkend erlebten sozialen Bedingungen auseinandersetzen. Identifizierte Widrigkeiten nehmen sie nicht einfach hin, sondern erarbeiten vielmehr Mittel, um als handelnde Subjekte die eigenen Umweltbeziehungen zu gestalten. Mit ihrem dialogischen Begegnungsformat eröffnen sich die Teilnehmenden eine (hier zunächst noch) gedankliche Erprobung strategischer Aneignung und damit tendenzielle „Wege aus der Bevormundung“ (Weinmann 2019, S. 179) von der sie umgebenden sozialen Umwelt.

Vor dem Hintergrund der teilnehmend beobachteten Online-Selbsthilfetreffen zeigt sich, dass die Verschränkung des Materiellen und des Virtuellen auch entlang atmosphärischer Dimensionen verläuft. Jener hergestellte atmosphärische Raum ist durch die vielfältigen zeitlichen und örtlichen Bezüge gekennzeichnet, welche die Teilnehmenden auf eine leiblich-affektive Weise trotz und geradezu wegen der physischen Distanz miteinander verbindet. Mit Blick auf die daraus resultierenden möglichen Impulse für sozialpädagogisches Handeln lässt sich zeigen, dass der atmosphärische Raum des Virtuellen auch als ein *Ort *fachlichen Handelns (Witzel 2021, S. 69) verstanden werden kann, sofern sozialpädagogisches Handeln nicht als nüchtern und distanziert, sondern als relational und gegenseitig verstanden und praktiziert wird. So kann einer viktimisierenden Kultur durch das Alltägliche, durch Erfahrungsaustausch, Parteilichkeit und Solidarität begegnet werden.

## Footnotes

1 VISION-RA (Video- und theatergestützte soziale Innovationen im Bereich recoveryförderlicher Arbeitsbündnisse in der (Gemeinde‑)Psychiatrie) ist ein hochschulübergreifendes Forschungsprojekt der Katholischen Hochschule Mainz und der Hochschule RheinMain Wiesbaden (Laufzeit: 01.08.2019 bis 31.07.2023).

2 Alle im Beitrag genannten Namen von Forschungssubjekten sind pseudonymisiert.

## References

[1] Deinet U, Reutlinger Ch, Bollweg P, Buchna J, Coelen Th, Otto H-U (2020). Aneignung. Handbuch Ganztagsausbildung.

[2] Ehlers C (2019). Stärken neu denken: die Kunst der Stärken fokussierten Zielarbeit in sozialen Handlungsfeldern.

[3] Goffman E (2018). Rahmen-Analyse. Ein Versuch über die Organisation von Alltagserfahrungen.

[4] Gugutzer R, Senge K, Schützeichel R (2013). Der Gefühlsraum. Hauptwerke der Emotionssoziologie.

[5] Haller F, Gräser H (2012). Selbsthilfegruppen. Konzepte, Wirkungen und Entwicklungen.

[6] Rockel F, Semmler T, Deutsche Arbeitsgemeinschaft Selbsthilfegruppen (DAG SHG) e. V. (2012). Erfahrungsbericht einer Selbsthilfegruppe für seelische Gesundheit. Selbsthilfegruppenjahrbuch.

[7] Schumacher B (2022). Ein gutes Leben macht Sinn – Was es braucht, um das Dasein als erfüllt zu erleben. Psychiatrische Pflege.

[8] Strauss AL, Corbin JM (1996). Grounded theory. Grundlagen qualitativer Sozialforschung.

[9] Weinmann S (2019). Die Vermessung der Psychiatrie: Täuschung und Selbsttäuschung eines Fachgebiets.

[10] Witzel M, Wunder M (2021). Sozialpädagogische Orte im digitalen Raum. Digitalisierung und Soziale Arbeit. Transformationen und Herausforderungen.

[11] Zwicknagel A (2022). Es muss auch anders gehen – Experienced Change-Making als weiterführender Schritt. Psychiatrische Pflege.

